# Amelogenesis imperfecta

**DOI:** 10.1186/1750-1172-2-17

**Published:** 2007-04-04

**Authors:** Peter JM Crawford, Michael Aldred, Agnes Bloch-Zupan

**Affiliations:** 1 Paediatric Dentistry, Division of Child Dental Health, Dental School, Lower Maudlin St., Bristol BS1 2LY, UK; 2 Dorevitch Pathology, Melbourne, Victoria, Australia; 3 Faculté de Chirurgie Dentaire, Université Louis Pasteur; Centre de référence des manifestations odontologiques des maladies rares, Centre Hospitalier Universitaire, Strasbourg, F-67000, France; 4 IGBMC (Institut de Génétique et de Biologie Moléculaire et Cellulaire), Département Génétique et Physiologie; Inserm, U596; CNRS, UMR7104, Illkirch, F-67400 France; 5 Eastman Dental Institute, Institute of Child Health, University College London, UK

## Abstract

Amelogenesis imperfecta (AI) represents a group of developmental conditions, genomic in origin, which affect the structure and clinical appearance of enamel of all or nearly all the teeth in a more or less equal manner, and which may be associated with morphologic or biochemical changes elsewhere in the body. The prevalence varies from 1:700 to 1:14,000, according to the populations studied. The enamel may be hypoplastic, hypomineralised or both and teeth affected may be discoloured, sensitive or prone to disintegration. AI exists in isolation or associated with other abnormalities in syndromes. It may show autosomal dominant, autosomal recessive, sex-linked and sporadic inheritance patterns. In families with an X-linked form it has been shown that the disorder may result from mutations in the amelogenin gene, *AMELX*. The enamelin gene, *ENAM*, is implicated in the pathogenesis of the dominant forms of AI. Autosomal recessive AI has been reported in families with known consanguinity. Diagnosis is based on the family history, pedigree plotting and meticulous clinical observation. Genetic diagnosis is presently only a research tool. The condition presents problems of socialisation, function and discomfort but may be managed by early vigorous intervention, both preventively and restoratively, with treatment continued throughout childhood and into adult life. In infancy, the primary dentition may be protected by the use of preformed metal crowns on posterior teeth. The longer-term care involves either crowns or, more frequently these days, adhesive, plastic restorations.

## Disease name

Amelogenesis imperfecta (AI) is a term for a clinically and genetically heterogeneous group of conditions that affect the dental enamel, occasionally in conjunction with other dental, oral and extraoral tissues.

## Definition and diagnosis criteria

AI represents a group of conditions, genomic in origin, which affect the structure and clinical appearance of the enamel of all or nearly all the teeth in a more or less equal manner, and which may be associated with morphologic or biochemical changes elsewhere in the body [1]. AI is a developmental condition of the dental enamel (characterised by hypoplasia and/or hypomineralisation) that shows autosomal dominant, autosomal recessive, sex-linked and sporadic inheritance patterns, as well as sporadic cases.

Diagnosis involves exclusion of extrinsic environmental or other factors, establishment of a likely inheritance pattern, recognition of phenotype and correlation with the dates of tooth formation to exclude a chronological developmental disturbance.

## Epidemiology

Reports vary widely depending upon the gene pool. Values of 1:14,000 in the USA [2] to 1:700 [3] have been reported.

## Classification

Many classifications of AI have evolved since the original division into hypoplastic and hypocalcified types in 1945 (See Table 1) [1,2,4-13]. Some have been exclusively based on the phenotype (appearance), others have used the phenotype as the primary discriminant and the mode of inheritance as a secondary factor in diagnosis.

**Table 1:** Classification systems applied to amelogenesis imperfecta

Weinmann *et al.*, 1945 [4]	*Two types based solely on phenotype: hypoplastic and hypocalcified*
Darling, 1956 [5]	*Five phenotypes based on clinical, microradiographic and histopathological findings*.
	Hypoplastic
	Group 1 – generalised pitting
	Group2 – vertical grooves (now known to be X-linked AI)
	Group 3 – Generalised hypoplasia
	Hypocalcified
	Type 4A – chalky, yellow, brown enamel
	Type 4B – marked enamel discolouration and softness with post-eruptive loss of enamel
	Type 5 – generalised or localised discolouration and chipping of enamel

Witkop, 1957 [6]	*Classification based primarily on phenotype. 5 types:*
	1. Hypoplastic
	2. Hypocalcification
	3. Hypomaturation
	4. Pigmented hypomaturation
	5. Local hypoplasia
	Added mode of inheritance as further means of delineating cases.

Schulze, 1970 [7]	*Classification based on phenotype and mode of inheritance*.

Witkop and Rao, 1971 [8]	*Classification based on phenotype and mode of inheritance. Three broad categories: hypoplastic, hypocalcificied, hypomaturation*.
	**a. **Hypoplastic
	Autosomal dominant hypoplastic-hypomaturation with taurodontism (subdivded into a and b according to author)
	Autosomal dominant smooth hypoplastic with eruption defect and resorption of teeth
	Autosomal dominant rough hypoplastic
	Autosomal dominant pitted hypoplastic
	Autosomal dominant local hypoplastic
	X-linked dominant rough hypoplastic
	**b. **Hypocalcified
	Autosomal dominant hypocalcified
	**c. **Hypomaturation
	X-linked recessive hypomaturation
	Autosomal recessive pigmented hypomaturation
	Autosomal dominant snow-capped teeth
	White hypomature spots?

Winter and Brook, 1975 [9]	*Classification based primarily on phenotype. Four main categories: hypoplasia, hypocalcification, hypomaturation, hypomaturation-hypoplasia with taurodontism, with mode of inheritance as a secondary means of sub-classification*.
	**a. **Hypoplasia
	Type I. Autosomal dominant thin and smooth hypoplasia with eruption defect and resorption of teeth
	Type II. Autosomal dominant thin and rough hypoplasia
	Type III. Autosomal dominant randomly pitted hypoplasia
	Type IV. Autosomal dominant localised hypoplasia
	Type V. X-linked dominant rough hypoplasia
	**b. **Hypocalcification
	Autosomal dominant hypocalcification
	**c. **Hypomaturation
	Type I. X-linked recessive hypomaturation
	Type II. Autosomal recessive pigmented hypomaturation
	Type III. Snow-capped teeth
	**d. **Hypomaturation-hypoplasia with taurodontism
	Type I. Autosomal dominant smooth hypomaturation with occasional hypoplastic pits and taurodontism
	Type II. Autosomal dominant smooth hypomaturation with thin hypoplasia and taurodontism

Witkop and Sauk, 1976 [2]	*Classification based on phenotype and mode of inheritance, similar to classification of Witkop and Rao (1971)*

Sundell and Koch, 1985 [10]	*Classification based solely on phenotype*

Witkop, 1988 [11]	*Four major categories based primarily on phenotype (hypoplastic, hypomaturation, hypocalcified, hypomaturation-hypoplastic with taurodontism) subdivided into 15 subtypes by phenotype and and secondarily by mode of inheritance*.
	Type I. Hypoplastic
	Type IA. Hypoplastic, pitted autosomal dominant
	Type IB. Hypoplastic, local autosomal dominant
	Type IC. Hypoplastic, local autosomal recessive
	Type ID. Hypoplastic, smooth autosomal dominant
	Type IE. Hypoplastic, smooth X-linked dominant
	Type IF. Hypoplastic, rough autosomal dominant
	Type IG. Enamel agenesis, autosomal recessive
	Type II. Hypomaturation
	Type IIA. Hypomaturation, pigmented autosomal recessive
	Type IIB. Hypomaturation, X-linked recessive
	Type IIC. Hypomaturation, snow-capped teeth, X-linked
	Type IID. Hypomaturation, snow-capped teeth, autosomal dominant?
	Type IIIA. Autosomal dominant
	Type IIIB. Autosomal recessive
	Type IV. Hypomaturation-hypoplastic with taurodontism
	Type IVA. Hypomaturation-hypoplastic with taurodontism, autosomal dominant
	Type IVB. Hypoplastic-hypomaturation with taurodontism, autosomal dominant

Aldred and Crawford, 1995 [12]	*Classification based on:*
	Molecular defect (when known)
	Biochemical result (when known)
	Mode of inheritance
	Phenotype

Hart *et al.*, 2002 [13]	*Proposed a molecular defect sub classification of the AMELX conditions*
	1.1 Genomic DNA sequence
	1.2 cDNA sequence
	1.3 Amino acid sequence
	1.4 Nucleotide and amino-acid sequences
	1.5 *AMELX *mutations described to date

Aldred *et al.*, 2003 [1]	*Classification based on:*
	Mode of inheritance
	Phenotype – Clinical and Radiographic
	Molecular defect (when known)
	Biochemical result (when known)

The multiplicity of classification systems based primarily or exclusively on phenotype can be confusing and it is not always possible to cross-reference between the various subtypes used, or to know which classification system might have been applied to a particular case. It would be ultimately useful to classify conditions by genome and by subsequent biochemistry [12,13]. More recently, it has been proposed that the mode of inheritance should be the primary mode of classification, with the phenotype as the secondary discriminant [1]. Although there are problems which arise from the pronounced genetic (inheritance) and phenotypic heterogeneity which can characterise AI, patients and practitioners do find an idea of likely inheritance to be useful and, in the absence of complete genomic and biochemical information, the "default" option of an initial classification by inheritance seems justified.

Diagnostic codes used in systems such as the International Classification of Diseases (ICD) are extremely limited in their application to AI, each having only a single code for the disorder as well as other abnormalities (*e.g. *ICD-9 520.5 for "hereditary disturbances in tooth structure not elsewhere classified: amelogenesis imperfecta, dentinogenesis imperfecta, odontogenesis imperfecta, dentinal dysplasia, shell teeth" without further subdivision and clearly with no distinction made according to either phenotype or mode of inheritance in each disorder). The Systematized Nomenclature in Medicine (SNOMED) records AI under a single numerical morphological category (M-23360) without taking into account the mode of inheritance and/or further phenotypic categorisation.

## Aetiology

Dental enamel is a highly mineralised tissue with over 95% of its volume occupied by unusually large, highly organised, hydroxyapatite crystals. The formation of this highly organised and unusual structure is thought to be rigorously controlled in ameloblasts through the interaction of a number of organic matrix molecules that include enamelin (*ENAM*; 4q21, OMIM *606585), amelogenin (*AMELX*; Xp22.3-p22.1, OMIM *300391), ameloblastin (*AMBN*; 4q21, OMIM *601259), tuftelin (*TUFT1*; 1q21, OMIM *600087), amelotin (*AMELOTIN *4q13) [14], dentine sialophosphoprotein (*DSPP*; 4q21.3, OMIM *125485), and a variety of enzymes such as kallikrein 4 (*KLK4*; 19q13.3–q13.4, OMIM *603767) and matrix metalloproteinase 20 (*MMP20*; 11q22.3–q23, OMIM *604629).

AI may be inherited in an X-linked manner or as an autosomal dominant or recessive trait. However, there are cases where the diagnosis of AI remains tentative in apparently sporadic cases of enamel defects. Ultimately, it is anticipated that molecular genetic tools will allow more precise diagnosis.

The different patterns of inheritance correspond with different genomic sites. Xp22.3 - p22.1 (*AMELX*, AIH1) is associated with the X-linked form. *AmelX *null mice have no amelogenin and display "distinctly abnormal teeth" with "disorganised, hypoplastic enamel" [15]. Another gene on the long arm of the X chromosome at the AIH3 locus must also be involved in enamel formation [16]. 4q11–q21 (AIH2, *ENAM *4q21) is associated with both autosomal dominant and autosomal recessive inheritance patterns and within this 4q13.3 has been identified as being associated with an autosomal recessive inheritance [17-20]. A transgenic animal model over-expressing ameloblastin has produced an AI-type phenotype [21]. Wang *et al.*, 2004 showed a relationship between the regulation of ameloblast differentiation and components of the bone morphogenetic protein (BMP) pathway [22]. This suggests that there is at least the potential for mutations in this pathway to account for some cases of amelogenesis imperfecta [22]. So far no mutation in the *amelotin *gene has been related to amelogenesis imperfecta [23].

## Clinical description

**Figure 1:**
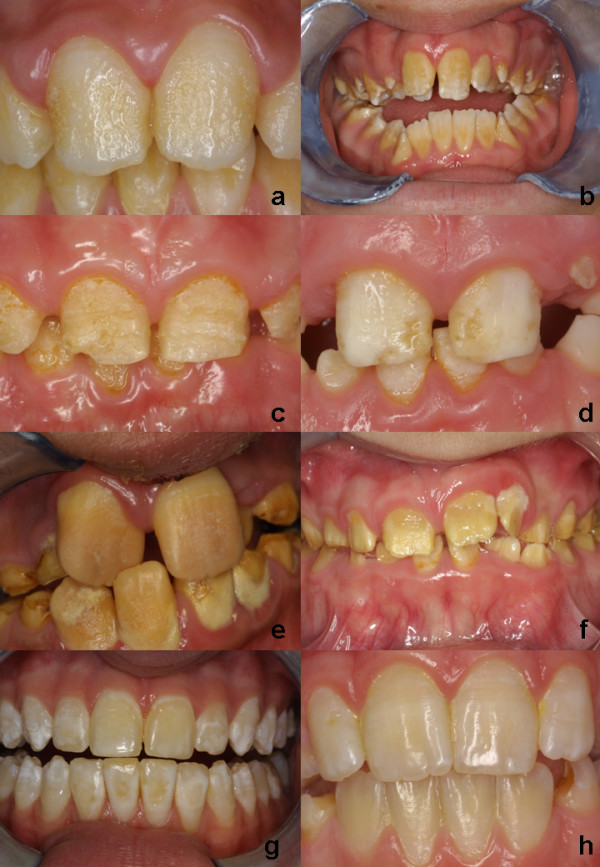
**Phenotypic descriptions of amelogenesis imperfecta**. Amelogenesis imperfecta may be subdivided at the clinical level into various forms depending on the type of defect and stages of enamel formation disturbed: hypoplastic (a, b, c, d), dysmineralised (e, f), hypomature (g, h). Note the pitted and ridged appearance of the enamel in a, the association of pitted enamel and open bite in b. c and d are slightly different phenotypic manifestations in a sister (showing a horizontal banding pattern) and brother. In the hypomineralised form (e and f) the enamel is rough, soft and discoloured. Amelogenesis imperfecta may be part of a syndrome as in f), a case of amelogenesis imperfecta and cone rod dystrophy. Various enamel defects (both hypoplastic and hypomineralised) may coexist in the same patient or even the same tooth (f). The hypomaturation forms (g, h) display an enamel of normal thickness and hardness but with a white-ish surface. They may be mistaken for fluorosis. (from A. Bloch-Zupan).

Amelogenesis imperfecta affects the enamel of all of the teeth of the affected individuals within a kindred, in a more or less equal manner, without reference to chronology, occasionally in association with other generalised conditions. The enamel may be hypoplastic, hypomineralised or both, and teeth affected may be discoloured, sensitive or prone to disintegration either post eruption (post-eruptive breakdown) or pre-eruption (idiopathic resorption) (Figure 1).

AI is a genetic disease that exists in isolation or associated to other symptoms in syndromes. It is either related to a single gene defect or arises from a microdeletion or chromosomal defects (Cone-rod dystrophy associated with AI, showing linkage to 2q11 is such an example [24,25]).

### X-linked forms of amelogenesis imperfecta

X-linked amelogenesis imperfecta (XAI) (OMIM #301200, Amelogenesis imperfecta 1, hypoplastic type, AIH1) shows the typical pattern of X-linked inheritance. Heterozygous females can pass on the mutant gene to children of either sex with the risk of this being 50%. The condition affects males and females in strikingly different ways. Males show the trait fully. They may have teeth which have only a thin layer of enamel of normal colour and translucency, or the enamel may be of normal thickness but poorly mineralised with loss of translucency and/or a yellow-brown discolouration. In some families, the phenotype appears to be both hypoplasia and abnormal mineralisation occurring together. When hypoplasia is the exclusive or predominant phenotype, there may be marked sensitivity of the teeth to thermal and osmotic stimuli. By contrast, females who inherit the mutant gene have vertical markings of the enamel as a result of X chromosome inactivation or Lyonisation. Thus, these heterozygous females may have teeth with vertical ridges and grooves as a result of hypoplasia of the enamel or have vertical bands of alternating normal and discoloured enamel [26].

The molecular basis of XAI has been established in some families. Positional cloning has demonstrated linkage of the disorder to the Xp22 region of the short arm of the X chromosome in some families and it has subsequently been shown that in these and other families the disorder is a result of mutations in the amelogenin gene *AMELX *[27-30]. Another locus on the long arm of the X chromosome has also been implicated in one family with XAI (AIH3) but this gene has not yet been cloned [16,31].

### The amelogenin gene (OMIM *300391)

The amelogenin gene *AMELX *maps to Xp22.3-p22.1 and codes for the amelogenin protein. It consists of seven exons spanning over 9 kilobases. Mutations reported include deletions of parts of the gene, single base mutations and premature stop codons. Certain parts of the gene may be critical in the control of enamel thickness, while other parts may play an important role in enamel mineralisation [32]. Alternative splicing of the amelogenin protein has been demonstrated but the clinical significance of this finding is yet to be determined. No evidence of pre-mutations has yet been found. Molecular analysis of the amelogenin gene may be helpful in confirming the diagnosis of XAI in particular cases, thereby allowing appropriate genetic counselling. There is no evidence of any mutations of the amelogenin gene *AMELY *(Yp11) on the Y chromosome (and the enamel forming role of genes on the Y chromosome and any mutations of these genes are as yet unknown, although it has been suggested that the Y site may contribute to tooth size [33]).

### Autosomal forms of amelogenesis imperfecta

Positional cloning has shown that the disorder in some families with autosomal dominant AI maps to 4q11–q21. The albumin *ALB*, ameloblastin *AMBN *and enamelin *ENAM *genes all map to the same region and are therefore candidate genes for autosomal AI. Tuftelin is another enamel protein, which maps differently to 1q21, and may be involved in other cases of autosomal AI. Mutations in genes coding for enamel proteases, which are involved in the degradation of enamel proteins, might also cause AI.

### Autosomal dominant amelogenesis imperfecta

Autosomal dominant AI (ADAI) typically affects one or more individuals in each generation of a family. There may be consistency in the clinical manifestations in every affected individual or there may be variable expression, resulting in substantial or subtle differences between different affected individuals in the same family.

The phenotype in ADAI may be predominantly or exclusively hypoplastic, manifested by thin enamel and spacing between the teeth, or in some pedigrees by rough, irregular or randomly pitted enamel. If the prime defect is in the amount of enamel matrix produced, the enamel will be hard, normally translucent and not subject to significant attrition. By contrast, in some individuals and families the phenotype may be predominantly or exclusively hypomineralised.

Some earlier classifications have subdivided these latter defects into hypocalcification defects, implying a more severe mineralisation abnormality, and hypomaturation defects, implying a lesser degree of hypomineralisation. At extremes, this subdivision is probably valid but in most cases it is difficult to be certain which would be the better descriptor, hence the use of the term dysmineralisation in recognition of the probable spectrum of defects of mineralisation of enamel in AI. Given the complexity of enamel formation, this is probably a realistic view until such time as the molecular and protein pathways of enamel formation have been elucidated. The most severe forms of dysmineralisation result in enamel that has a "cheesy" consistency and is easily lost, resulting in teeth of reduced size and considerable sensitivity.

Some cases will show both enamel hypoplasia and dysmineralisation. Thus, in a number of individuals, the teeth will be reduced in size *ab initio *with thinner enamel, small crowns and spacing as well as showing a yellowish-brown hue as evidence of a coincidental mineralisation defect.

### *ENAM *4q21

The enamelin gene (*ENAM*) has been mapped to chromosome 4q, to the same region as AIH2 (Amelogenesis imperfecta 2, hypoplastic local, AIH2, OMIM #104500). Rajhpar *et al. *have described an extensive family in which the causative gene mapped to 4q11–q21 [18].

Kida *et al. *reported an extensive Japanese family with an apparently similar phenotype, whose condition was linked to "a single-G deletion within a series of 7 G residues at the exon 9-intron 9 boundary of the enamelin gene" in this region [19]. A local, hypoplastic form of the autosomal dominant inherited condition was traced by Mardh *et al. *[20] to a nonsense mutation in the enamelin gene. This gave rise to a truncated protein which was expressed as the mentioned local hypoplasia, rather than a generalised form seen with a "splice-site" mutation. The work of Kim *et al. *further relates phenotype to genotype and identifies a mid-crown, horizontal form of genetically determined hypoplasia [34].

### Chromosome 8q24.3

An additional locus for autosomal dominant amelogenesis imperfecta has been found recently on chromosome 8q24.3 [35].

### Autosomal recessive amelogenesis imperfecta

Autosomal recessive AI (ARAI) should be considered if there is known consanguinity in a family with an affected individual. This may be more often encountered in certain ethnic and cultural groups where intermarriage within the family may be more common (for example, AI in association with cone rod dystrophy, a syndromic condition [24,36]). ARAI will also be more prevalent where there is a high frequency of the mutant gene in a population, such as in some Polynesian communities [37,38].

### *ENAM *4q21

Amelogenesis imperfecta, hypoplastic, and openbite malocclusion, OMIM #608563; Hypoplastic enamel pitting, localized, included #608563.

Most interestingly, Hart *et al. *(2003) described three probands with ostensibly autosomal recessive AI [17]. They found that where the individuals were homozygous for the g.13185_13186insAG *ENAM *mutation on chromosome 4, they showed enamel pitting with an anterior open bite, whereas heterozygosity presented only with the enamel pitting. They described this as "dose dependency". Enamel phenotypes of *ENAM *mutations may be dose-dependent, with generalised hypoplastic AI segregating as a recessive trait and localised enamel pitting segregating as a dominant trait [39].

Other authors associated with the same centre presented an extensive survey at the clinical, familial, radiographic, microscopic and protein analysis level of nine Jordanian families with ARAI. They found a wide diversity of phenotypes. Whilst supporting the principle of classification first by inheritance pattern, they suggested the existence of four clear sub-types of ARAI [40].

### KALLIKREIN 4

The first mutation in the kallikrein gene family, the *KLK4 *gene (that maps to chromosome 19q13.4), has recently been identified as being associated with autosomal recessive hypomaturation amelogenesis imperfecta [41].

### MMP-20

A mutation in the matrix metalloproteinase 20 gene (*MMP-20*) in the region 11q22.3–q23 has been described as being associated with autosomal recessive pigmented hypomaturation amelogenesis imperfecta [42,43].

### Sporadic cases of amelogenesis imperfecta

Apparently sporadic cases of AI may have one of several causes. They may represent examples of ARAI, or they may be due to new mutations, or they may be illustrative of variable expression with or without incomplete penetrance of a dominant gene. Careful examination of other family members is vital in such instances, and it is important to avoid inappropriate classification of individuals as AI if, in fact, their tooth abnormality is due to non-genetic causes (such as fluorosis or tetracycline staining). We do not yet know whether new mutations will be passed on as an autosomal dominant trait, though evidence from other genetic disorders would suggest this to be a likely scenario.

Of recent years, a condition referred to as Molar-Incisor Hypomineralisation (MIH) has become prominent [44,45]. The aetiology of this condition, which affects one or more of the first permanent molars in a chronologically-reminiscent but unrelated pattern, together with a seemingly random number of permanent anterior teeth, is unknown. There are reports of affected sibs [46]. MIH is not presently classified as amelogenesis imperfecta.

For recent review on the molecular genetics of AI see [47,48].

### Syndromes including amelogenesis imperfecta

Earlier strict definitions of AI have specified an enamel defect without the involvement of other structures. However, there is an intellectual problem in adopting too narrow a definition and such a restriction is probably counterproductive as far as families are concerned.

AI is a genetic disease that may exist either in isolation or associated to or with other features in syndromes. It is either related to a single gene defect or arises from a microdeletion or chromosomal defect. AI, showing linkage to 2q11 and associated with cone-rod dystrophy, is such an example [24,25].

### Amelogenesis imperfecta a syndrome in itself

Clinically, a skeletal anterior open bite is seen in approximately 50% of patients with AI of either X-linked or autosomal inheritance. Such an association might be regarded as a syndrome but this does not appear as such in any classification. The significance of this common association has yet to be elucidated.

Predominantly phenotypic classifications of AI have included a variant with taurodontism as an intrinsic feature – AI with taurodontism (AIT) (OMIM #104510). This also goes beyond the strict definition of AI yet it is reasonable to include the condition in any classification of AI given that taurodontism is regarded as an ectodermal trait. The sheath of Hertwig, that maps the shape of the roots of teeth, is a derivative of the enamel organ and is also responsible for differentiation of the inner dental epithelial cells to ameloblasts producing enamel proteins. The subtle dentine changes reported by Winter *et al. *(1969) add further to our difficulties in understanding the complexities involved in some cases [49].

### Amelogenesis imperfecta in syndromes

Less clearly, there are strong similarities between AIT and the tricho-dento-osseous (TDO) syndrome (OMIM #190320), which has the additional features of "curly hair" and skeletal changes including bone sclerosis. While the hair changes might represent a common ectodermal defect in TDO, the bony changes are more difficult to explain *via *a common pathway, as this assumes a mesodermal defect. TDO is caused by a mutation in the *DLX3 *gene [50]. One molecular study has reported that AIT and TDO are genetically distinct [51], whereas a later paper suggests that TDO and Amelogenesis imperfecta hypoplastic-hypomaturation with taurodontism (AIHHT) are allelic for *DLX3 *[52].

The literature records further examples of seemingly AI-like changes associated with other whole body findings, hitherto excluded from a diagnosis of AI. If we accept that AI may occur as an isolated trait, but also in association with a range of other abnormalities, then many different syndromes need to be considered in the differential diagnosis of patients with enamel defects. For further information regarding the full range of symptoms associated with AI, the reader is referred to Online Mendelian Inheritance in Man (For example see Kohlschutter syndrome, OMIM %226750; Platyspondyly with amelogenesis imperfecta, OMIM 601216; Amelogenesis imperfecta and nephrocalcinosis, OMIM 204690; cone rod dystrophy and amelogenesis imperfecta, OMIM %217080).

The published association of hypoplastic amelogenesis imperfecta with nephrocalcinosis may raise the question of the need for renal examination when a diagnosis of AI is given [53].

### Enamel defects but not Amelogenesis imperfecta in syndromes

Differential diagnosis of the causes of enamel defects is important for both therapeutic, professional and patient-personal reasons. There are many alternative causes of enamel defects but, for example, a localised defect of a central incisor, coupled with the non-eruption of an adjacent tooth, may point to an injury in childhood and possible damage to the unerupted tooth. Rarely, the recognition of regional odontodysplasia, a rare developmental abnormality of all three dental tissues, enamel, dentine and pulp affecting a segment of the dentition, will assist in its management, which has all too often previously condemned the teeth to extraction [54].

Many persons affected by these conditions are concerned for the teeth of their children. A careful diagnosis, particularly in relation to inheritance, will be important to many such affected families.

## Diagnostic methods

### Clinical

The family history, pedigree plotting, clinical observation and meticulous recording form the backbone of diagnosis in this, as in any potentially inherited condition. Extra-oral radiographs may reveal the presence of unerupted and sometimes spontaneously resorbing teeth. Intra-oral radiographs will reveal the relative contrast between enamel and dentine in cases where mineralisation may have been affected. The same films in conjunction with clinical observation will provide information on the degree of any enamel hypoplasia.

### Genetic diagnosis

Laboratory genetic diagnosis is presently only a research tool.

## Differential diagnosis

Extrinsic disorders of tooth formation, chronological disorders of tooth formation and localised disorders of tooth formation should be considered in the differential diagnosis.

The teeth form a record of dysplasia (abnormal growth). Dental enamel does not "heal" – other than at the microscopic level in the context of early demineralisation caused by caries. As a result, many disturbances of metabolism now resolved, as well as genetic conditions, will be reflected in the appearance of the teeth. Four simple questions are valuable here – does anyone else in the family have anything like this; are all of the teeth affected in a similar manner; is there a chronological distribution to the appearance seen; is there anything in the past medical history which might have caused sufficient metabolic disturbance to affect enamel formation – and form the foundation for differential diagnosis.

The commonest differential diagnosis is dental fluorosis. The variability of this condition, from mild white "flecking" of the enamel to profoundly dense white colouration with random, disfiguring areas of staining and hypoplasia, requires careful questioning to distinguish from AI. Fluorosis may present with areas of horizontal white banding corresponding to periods of more intense fluoride intake and may show the premolars or second permanent molars to be spared (chronological distribution). In the latter case, the history will often reveal excessive fluoride intake either in terms of a habit such as eating toothpaste in childhood, or related to a local water supply.

A similar distribution of findings – chronological enamel hypoplasia – can arise from one of many causes during the time of tooth formation. Ranging from gastrointestinal upset of a prolonged nature, such as coeliac disease (a diagnosis which may not be confirmed until later life), to anti-leukaemic therapy; these causes may be identified from the history and from the chronological distribution of the markings seen.

Molar-Incisor Hypomineralisation (MIH), which shows features quite unlike these findings, has been considered above.

## Genetic counselling

There is a growing acceptance that a classification of (inherited) enamel defects based primarily or exclusively on phenotype (appearance) is problematical. For this reason, the mode of inheritance and underlying genomic change are as important or more important discriminators. This is particularly so in relation to genetic counselling of affected individuals and their families. With the clearly stated proviso that genetic heterogeneity may make difficulties for any discussion of Mendelian inheritance, but with increasing access to molecular identification, families greatly appreciate discussion of likely risk and future inheritance. These conditions are often embarrassing, distressing and lead to social exclusion and ridicule. Sensitive interview and early supportive intervention are essential.

## Treatment

The supportive clinical care needed by these individuals is substantial both in terms of clinical and emotional demands. Patients have been known to cover their teeth with pieces of paper, chewing gum or other materials in order to mimic an "ordinary" appearance. At least one individual stole to fund his dental care [55]. Adolescents in particular have been known to become reclusive and withdrawn, and even to threaten suicide because of their disfigured teeth. Many young people with AI request the removal of their teeth and the fitting of dentures in a society where having one's own teeth is the longed-for norm.

Treatment is as ever based on the principles of prevention before intervention. However, in these patients' cases, intervention will likely be earlier and more radical than for others.

The progression of treatment during childhood has been described as a temporary phase followed by a transitory phase [56]. In infancy, the primary dentition is protected by the use of preformed metal crowns on posterior teeth. Either polycarbonate crowns or composite restorations are used on anterior teeth. Whilst it is to be hoped that such restorations may be placed using local anaesthetic, either behavioural considerations, or rapid wear of the teeth, may force a decision to be made to use some form of altered consciousness in young or fearful children [57]. If general anaesthesia is contemplated, this is a difficult decision for many parents and operators. It is little consolation whilst facing such a decision that delay might itself lead to the need for a similar decision in order to remove decayed and painful teeth, which might otherwise have been useful and aesthetic until the normal time of shedding.

The eruption of the permanent dentition, beginning at six years of age, presents a particularly difficult period. Some of the forms of AI present with hypersensitive teeth or with teeth that crumble, and both presentations provide a very real disincentive to good oral hygiene and are very difficult to restore. Those cases with enamel which is reasonably hard (*i.e. *less hypomineralised) and thin (*i.e. *more hypoplastic) lend themselves fairly readily to the use of preformed metal crowns on posterior teeth, as they erupt and composite restorations on anterior teeth. These latter may need to be added to as more of the cervical part of the tooth is revealed. Restorative treatment requires local analgesia at least.

Children with AI are not without malocclusions and it is important that a restorative dentist and an orthodontist are involved with the paediatric dentist in the care plan from the child's early age. It is the paediatric dentist's role to deliver to the (adult) restorative dentist a patient who is motivated, with good oral care practices and with no treatment option compromised by previous activity. The anterior open bite seen in some cases of AI requires consideration of surgical as well as restorative management.

The longer-term care still revolves around either crowns or, more frequently these days, adhesive, plastic restorations. However, whilst many practitioners strive rightly to delay the first "tooth-cutting" restoration, conversations with a substantial number of adults with AI suggest that this professional restraint may be unwelcome and paternalistic. Some of these same adults will recount that, if they had realised that restored teeth must eventually fail, they would have chosen tooth-tissue destructive, but aesthetically more attractive restorations earlier in their adolescence, in order to appear most "ordinary" to their peers at an important time in social development.

## References

[B1] Aldred MJ, Crawford PJM, Savarirayan R (2003). Amelogenesis imperfecta – a classification and catalogue for the 21st century. Oral Dis.

[B2] Witkop CJ, Sauk JJ, Stewart R, Prescott G (1976). Heritable defects of enamel. Oral Facial Genetics.

[B3] Backman B, Holm AK (1986). Amelogenesis imperfecta: prevalence and incidence in a northern Swedish county. Community Dent Oral Epidemiol.

[B4] Weinmann JP, Svoboda JF, Woods RW (1945). Hereditary disturbances of enamel formation and calcification. J Am Dent Assoc.

[B5] Darling AI (1956). Some observations on amelogenesis imperfecta and calcification of the dental enamel. Proc Roy Soc Med.

[B6] Witkop CJ (1957). Hereditary defects in enamel and dentin. Prosc First Cong Human Genet Acta Genetica Statist Med.

[B7] Schultze C, Gorlin RJ, Goldman HM (1970). Developmental abnormalities of teeth and jaws. Thoma's oral pathology.

[B8] Witkop CJ, Rao S, Bergsma E (1971). Inherited defects in tooth stucture. The clinical delineation of birth defects Part XI orofacial structures.

[B9] Winter GB, Brook AH (1975). Enamel hypoplasia and anomalies of the enamel. Dent Clin North Am.

[B10] Sundell S, Koch G (1985). Hereditary amelogenesis imperfecta. I. Epidemiology and clinical classification in a Swedish child population. Swed Dent J.

[B11] Witkop CJ (1988). Amelogenesis imperfecta, dentinogenesis imperfecta and dentin dysplasia revisited: problems in classification. J Oral Pathol.

[B12] Aldred MJ, Crawford PJM (1995). Amelogenesis imperfecta – towards a new classification. Oral Diseases.

[B13] Hart PS, Hart TC, Simmer JP, Wright JT (2002). A nomenclature for X-linked amelogenesis imperfecta. Arch Oral Biol.

[B14] Iwasaki K, Bajenova E, Somogyi-Ganss E, Miller M, Nguyen V, Nourkeyhani H, Gao Y, Wendel M, Ganss B (2005). Amelotin-a Novel Secreted, Ameloblast-specific Protein. J Dent Res.

[B15] Gibson CW, Yuan ZA, Hall B, Longenecker G, Chen E, Thyagarajan T, Sreenath T, Wright JT, Decker S, Piddington R, Harrison G, Kulkarni AB (2001). Amelogenin-deficient mice display an amelogenesis imperfecta phenotype. J Biol Chem.

[B16] Aldred MJ, Crawford PJ, Roberts E, Gillespie CM, Thomas NS, Fenton I, Sandkuijl LA, Harper PS (1992). Genetic heterogeneity in X-linked amelogenesis imperfecta. Genomics.

[B17] Hart TC, Hart PS, Gorry MC, Michalec MD, Ryu OH, Uygur C, Ozdemir D, Firatli S, Aren G, Firatli E (2003). Novel ENAM mutation responsible for autosomal recessive Amelogenesis imperfecta and localised enamel defects. J Med Genet.

[B18] Rajpar MH, Harley K, Laing C, Davies RM, Dixon MJ (2001). Mutation of the gene encoding the enamel-specific protein, enamelin, causes autosomal-dominant amelogenesis imperfecta. Hum Mol Genet.

[B19] Kida M, Ariga T, Shirakawa T, Oguchi H, Sakiyama Y (2002). Autosomal-dominant hypoplastic form of amelogenesis imperfecta caused by an enamelin gene mutation at the exon-intron boundary. J Dent Res.

[B20] Mardh CK, Backman B, Holmgren G, Hu JC, Simmer JP, Forsman-Semb K (2002). A nonsense mutation in the enamelin gene causes local hypoplastic autosomal dominant amelogenesis imperfecta (AIH2). Hum Mol Genet.

[B21] Paine ML, Wang HJ, Luo W, Krebsbach PH, Snead ML (2003). A transgenic animal model resembling amelogenesis imperfecta related to ameloblastin overexpression. J Biol Chem.

[B22] Wang X-P, Suomalainen M, Jorgez CJ, Matzuk MM, Werner S, Thesleff I (2004). Follistatin regulates enamel patterning in mouse incisors by asymmetrically inhibiting BMP signaling and ameloblast differentiation. Dev Cell.

[B23] Santos MC, Hart PS, Ramaswami M, Kanno CM, Hart TC, Line SR (2007). Exclusion of known gene for enamel development in two Brazilian families with amelogenesis imperfecta. Head Face Med.

[B24] Jalili IK, Smith NJD (1988). A progressive cone-rod dystrophy and amelogenesis imperfecta: a new syndrome. J Med Genet.

[B25] Downey LM, Keen TJ, Jalili IK, McHale J, Aldred MJ, Robertson SP, Mighell A, Fayle S, Wissinger B, Inglehearn CF (2002). Identification of a locus on chromosome 2q11 at which recessive amelogenesisimperfecta and cone-rod dystrophy cosegregate. Eur J Hum Genet.

[B26] Crawford PJM, Aldred MJ (1992). X-linked amelogenesis imperfecta: presentation of two kindreds and a review of the literature. Oral Surg Oral Med Oral Pathol.

[B27] Lagerstrom M, Dahl N, Nakahori Y, Nakagome Y, Backman B, Landegren U, Pettersson U (1991). A deletion in the amelogenin gene (AMG) causes X-linked amelogenesis imperfecta (AIH1). Genomics.

[B28] Lagerstrom-Fermer M, Nilsson M, Backman B, Salido E, Shapiro L, Pettersson U, Landegren U (1995). Amelogenin signal peptide mutation: correlation between mutations in the amelogenin gene (AMGX) and manifestations of X-linked amelogenesis imperfecta. Genomics.

[B29] Aldred MJ, Crawford PJM, Roberts E, Thomas NST (1992). Identification of a nonsense mutation in the amelogenin gene (AMELX) in a family with X-linked amelogenesis imperfecta (AIH1). Hum Genet.

[B30] Lench NJ, Winter GB (1995). Characterisation of molecular defects in X-linked amelogenesis imperfecta (AIH1). Hum Mutat.

[B31] Crawford PJ, Aldred MJ (1993). Clinical features of a family with X-linked amelogenesis imperfecta mapping to a new locus (AIH3) on the long arm of the X chromosome. Oral Surg Oral Med Oral Pathol.

[B32] Wright JT, Hart PS, Aldred MJ, Seow K, Crawford PJ, Hong SP, Gibson CW, Hart TC (2003). Relationship of phenotype and genotype in X-linked amelogenesis imperfecta. Connect Tissue Res.

[B33] Alvesalo L, Portin P (1980). 47, XXY males: sex chromosomes and tooth size. Am J Hum Genet.

[B34] Kim JW, Seymen F, Lin BP, Kiziltan B, Gencay K, Simmer JP, Hu JC (2005). ENAM mutations in autosomal-dominant amelogenesis imperfecta. J Dent Res.

[B35] Mendoza G, Pemberton TJ, Lee K, Scarel-Caminaga R, Mehrian-Shai R, Gonzalez-Quevedo C, Ninis V, Hartiala J, Allayee H, Snead ML, Leal SM, Line SR, Patel PI (2007). A new locus for autosomal dominant amelogenesis imperfecta on chromosome 8q24.3. Hum Genet.

[B36] Michaelides M, Bloch-Zupan A, Holder GE, Hunt DM, Moore AT (2004). An autosomal recessive cone-rod dystrophy associated with amelogenesis imperfecta. J Med Genet.

[B37] Crooks MC (1990). Prevalence of developmental defects of enamel in children and young adults in the Cook Islands. N Z Dent J.

[B38] Smillie AC, Rodda JC, Kawasaki K (1986). Some aspects of hereditary defects of dental enamel, including some observations on pigmented Polynesian enamel. N Z Dent J.

[B39] Ozdemir D, Hart PS, Firatli E, Aren G, Ryu OH, Hart TC (2005). Phenotype of ENAM mutations is dosage-dependent. J Dent Res.

[B40] Nusier M, Yassin O, Hart TC, Samimi A, Wright JT (2004). Phenotypic diversity and revision of the nomenclature for autosomal recessive amelogenesis imperfecta. Oral Surg Oral Med Oral Pathol Oral Radiol Endod.

[B41] Hart PS, Hart TC, Michalec MD, Ryu OH, Simmons D, Hong S, Wright JT (2004). Mutation in kallikrein 4 causes autosomal recessive hypomaturation amelogenesis imperfecta. J Med Genet.

[B42] Kim JW, Simmer JP, Hart TC, Hart PS, Ramaswami MD, Bartlett JD, Hu JC (2005). MMP-20 mutation in autosomal recessive pigmented hypomaturation Amelogenesis imperfecta. J Med Genet.

[B43] Ozdemir D, Hart PS, Ryu OH, Choi SJ, Ozdemir-Karatas M, Firatli E, Piesco N, Hart TC (2005). MMP20 active-site mutation in hypomaturation amelogenesis imperfecta. J Dent Res.

[B44] Weerheijm KL (2003). Molar incisor hypomineralisation (MIH). Eur J Paediatr Dent.

[B45] Weerheijm KL, Duggal M, Mejare I, Papagiannoulis L, Koch G, Martens LC, Hallonsten AL (2003). Judgement criteria for molar incisor hypomineralisation (MIH) in epidemiologic studies: a summary of the European meeting on MIH held in Athens, 2003. Eur J Paediatr Dent.

[B46] Crawford PJM, Aldred MJ (2005). Paediatric Dentistry.

[B47] Stephanopoulos G, Garefalaki M-E, Lyroudia K (2005). Genes and related proteins involved in amelogenesis imperfecta. J Dent Res.

[B48] Wright JT (2006). The molecular etiologies and associated phenotypes of amelogenesis imperfecta. Am J Med Genet A.

[B49] Winter GB, Lee KW, Johnson NW Hereditary amelogenesis imperfecta. A rare autosomal dominant type. Br Dent J.

[B50] Price JA, Bowden DW, Wright JT, Pettenati MJ, Hart TC (1998). Identification of a mutation in DLX3 associated with tricho-dento-osseous (TDO) syndrome. Hum Mol Genet.

[B51] Price JA, Wright JT, Walker SJ, Crawford PJ, Aldred MJ, Hart TC (1999). Tricho-dento-osseous syndrome and amelogenesis imperfecta with taurodontism are genetically distinct conditions. Clin Genet.

[B52] Dong J, Amor D, Aldred MJ, Gu T, Escamilla M, MacDougall M (2005). DLX3 mutation associated with autosomal dominant amelogenesis imperfecta with taurodontism. Am J Med Genet A.

[B53] Hunter L, Addy LD, Knox J, Drage N (2007). Is amelogenesis imperfecta an indication for renal examination?. Int J Paediatr Dent.

[B54] Crawford PJ, Aldred MJ (1989). Regional odontodysplasia: a bibliography. J Oral Pathol Med.

[B55] Aldred MJ, Crawford PJM, Savarirayan R, Savulescu J (2003). It's only teeth – is there a limit to genetic testing?. Clinical Genet.

[B56] Bouvier D, Duprez JP, Bois D (1996). Rehabilitation of young patients with amelogenesis imperfecta: a report of two cases. ASDC J Dent Child.

[B57] Sapir S, Shapira J (2001). Dentinogenesis imperfecta: an early treatment strategy. Pediatr Dent.

